# A new lineage of looper moths discovered in the South African Cape Floristic Region (Lepidoptera, Geometridae)

**DOI:** 10.3897/zookeys.1267.174407

**Published:** 2026-01-30

**Authors:** Mikael Englund, Elina Laiho, Kyung Min Lee, Hermann Staude, Max Söderholm, Pasi Sihvonen

**Affiliations:** 1 Zoology Unit, Finnish Museum of Natural History, University of Helsinki, Helsinki, Finland University of Helsinki Helsinki Finland; 2 Lep Soc Africa, George, South Africa Lep Soc Africa George South Africa

**Keywords:** Biogeography, geometrid moths, integrative taxonomy, micro-CT imaging, molecular phylogeny, new genus, new species, scanning electron microscopy

## Abstract

The Cape Floristic Region (CFR) of South Africa is globally recognized for its exceptional biodiversity and endemism, but despite extensive floral studies, its phytophagous insect fauna remains poorly studied. Here, we employ an integrative taxonomic approach including macro- and micro-photography, micro-CT scanning, scanning electron microscopy, and multigene molecular phylogenetics to describe a recently discovered, morphologically and genetically distinct lineage of geometrid moths (Lepidoptera: Geometridae) from the CFR, comprising a new genus, *Fynbosia* Englund, Staude & Sihvonen, **gen. nov**. and two new species, *F. horingaria* Englund, Staude & Sihvonen, **sp. nov**. and *F. unicaria* Englund, Staude & Sihvonen, **sp. nov**. Morphological and molecular evidence support the placement of *Fynbosia***gen. nov**. within the subfamily Larentiinae but suggest no close affiliation to any described tribe. The new genus appears to be endemic to the CFR’s montane fynbos and renosterveld vegetation types, which may act as ecological islands that foster speciation. The discovery underscores the overlooked insect diversity of the region and the urgent need for more comprehensive surveys. Our findings contribute to a better understanding of geometrid diversity and highlight the value of integrative taxonomy and non-destructive imaging in documenting rare and cryptic lineages.

## Introduction

**Figure 1.**
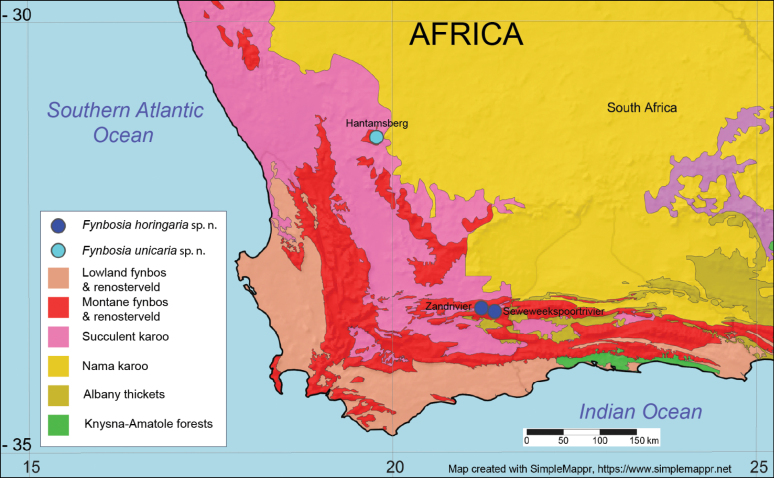
The Cape Floral Region and the known locations of *Fynbosia* moths. Map base and vegetation type limits retrieved from https://www.simplemappr.net.

The Cape Floristic Region (CFR, Fig. 1) in the southwestern tip of Africa is recognized as one of the six floral kingdoms of the Earth with a high biodiversity and highly endemic flora and fauna (Born et al. 2007; Grobler and Cowling 2021; Samways et al. 2024a). Almost 11 500 species of plants exist in the CFR, of which more than three-quarters are endemic (Forest et al. 2018). The CFR can be divided into several biomes, each subdivided into several subtypes (e.g., Mucina and Rutherford 2006, Fig. 1). Due to the high floral endemism and stable geological history, the region also harbours a high but incompletely studied phytophagous insect diversity (Procheş and Cowling 2006; Kemp and Ellis 2017; Samways et al. 2024a). Several undescribed species of geometrid moths from the CFR are present in extant insect collections, and we discovered two unknown male moth specimens in the Western and Northern Cape provinces, some 300 kilometres apart, in March and April of 2022. These specimens turned out to belong to two closely related undescribed species representing an isolated lineage that we classify in the moth family Geometridae and subfamily Larentiinae.

We used an integrative approach (Padial et al. 2010) for species delimitation. i.e., the validity of a species is more probable if supported by several unlinked character sets widely used in geometrid taxonomy (see e.g., Hausmann 2001; Sihvonen 2005; Müller et al. 2019) such as external and genitalia morphology, mitochondrial DNA barcode and distribution. Here, we present an integrative taxonomy of the new taxa and describe the genus *Fynbosia* (gen. nov.) and two species *F. horingaria* (sp. nov.) and *F. unicaria* (sp. nov.) as new to science.

## Materials and methods

**Figure 2.**
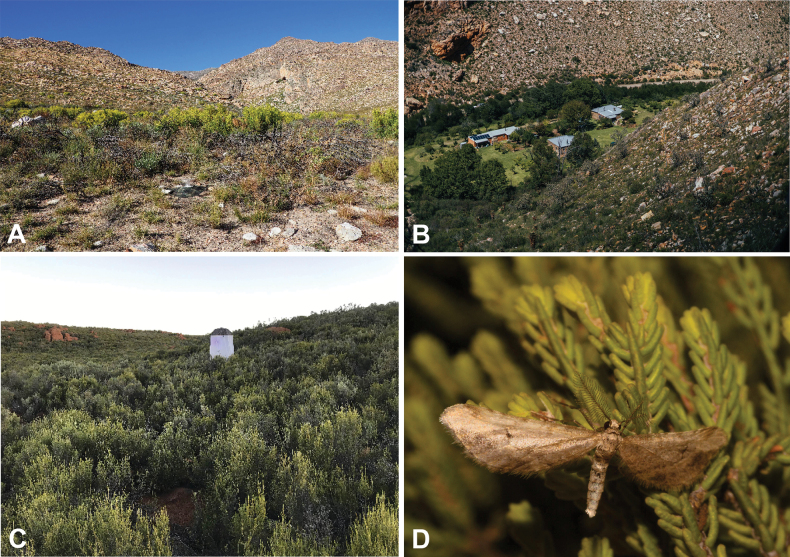
Habitats of *Fynbosia* moths: *Fynbosia horingaria* sp. nov., habitats from Zandrivier (**A**) and Seweweekspoortrivier (**B**) near Calitzdorp, South Africa; habitat of *Fynbosia unicaria* sp. nov., on Hantamsberg near Calvinia, South Africa (**C**); live *Fynbosia* male specimen (**D**). Photo (**B**) retrieved from https://www.facebook.com/7weekspoort/.

**Figure 3.**
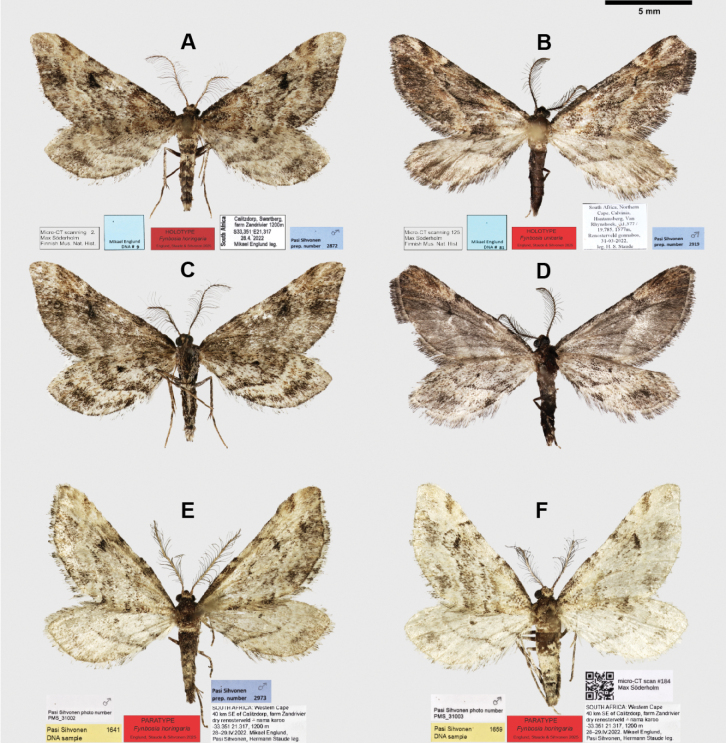
Adult male specimens of *Fynbosia*. *Fynbosia horingaria* sp. nov., holotype (**A, C, E, F**): **A**. Dorsal view; **C**. Ventral view; **E, F**. Paratypes dorsal view. *Fynbosia unicaria* sp. nov., holotype (**B, D**): **B**. Dorsal view; **D**. Ventral view.

During an expedition to the South African Swartberg Mountain Range in late April 2022, we found a small, rather worn geometrid moth individual attracted to the dim light of our bathroom window in the farm Zandrivier near Calitzdorp (Figs 1, 2A, 3A, C). Since none of the authors (ME, HS, PS) could recognize the specimen on site, we took it with us for further examination to Helsinki, Finland.

Later, Hermann Staude recognised that he had collected a rather similar appearing geometrid male moth from the Hantamsberg near Calvinia, Northern Cape, South Africa (Figs 1, 2C, 3B, D) some four weeks earlier. This specimen turned out to belong to a related, but different species.

In mid-April of 2023, Axel Hofmann and Jörg-Uwe Meinecke collected three more specimens, two males and a female of the first-mentioned species from Staude’s light trap set up in Seweweekspoortrivier, near Calitzdorp (Figs 1, 2B), some four kilometres southeast of the Zandrivier farm, where the first specimen was found. Unfortunately, the female specimen has not been located for closer scrutiny. We discovered a further two males (Fig. 3E, F) in 2025 from material collected in 2022 at Zandrivier Farm by the authors.

Authors (HS, ME, PS), Jannik Wagner (JW) of the State Museum of Natural History Stuttgart (SMNS), and Axel Hausmann (AH) of the Bavarian State Collection of Zoology in Munich (ZSM), examined the Lepidoptera collections of Ditsong Natural History Museum, Pretoria, South Africa (DMNH; HS, ME, JW), Natural History Museum in London (NHMUK; PS), and ZSM (AH, PS) for additional specimens similar to those we had discovered but found none. Consequently, we are aware of just seven specimens belonging to this undescribed moth lineage, and we had four male specimens (Fig. 3) from 2022 available for closer examination and description. All specimens were collected under the relevant permits held by Hermann Staude.

All specimens were attracted to artificial light at night: the *F. horingaria* sp. nov. holotype to a standard room light and the rest to battery-operated UV bulbs placed inside a custom-made light trap (Fig. 2C).

The holotype specimens were photographed using a Canon digital camera (model: EOS 5D) with an EF 100 mm macro lens attached to a StackShot automated macro rail, and the resulting multifocal images stacked in Zerene Stacker software (v. 1.04) (Figs 3, 6). The stacked image files were edited in Adobe Photoshop (v. 25.4.0), and the final plates were arranged using CorelDraw (v. 25.1.0) and Adobe Illustrator (v. 28.1).

The type specimens were scanned using a Nikon micro-CT scanner (model: XT H 225) at the Finnish Museum of Natural History (Luomus, University of Helsinki) and rendered images along with the videoclips (Fig. 5, Suppl. materials 1, 2) following a non-destructive protocol we have previously described in detail (Englund et al. 2024).

After detaching an antenna from the paratype of *Fynbosia horingaria* sp. nov. (Fig. 3F), it was cut into two pieces. The proximal part was attached dorsal side up, and the distal part ventral side up with two-sided tape on a metal pod for scanning electron microscopy (SEM) imaging. The preparates were platinum-coated and scanned with Zeiss Gemini device (model: 460 FEG) in the University of Helsinki Institute of Biotechnology Electron Microscopy Unit (Fig. 7).

Following imaging, we removed the abdomens from the holotypes, dissected the genitalia using established preparation protocols (e.g., Hardwick 1950; Robinson 1976; Sihvonen 2001, 2005) stained the preparates with Chlorazol Black, and after photography, mounted them in Euparal. The structural characters of the male genitalia were photographed during dissection through a Leica stereo microscope (model: DM1000 LED) and an attached Leica camera (model: MC170 HD) for scrutiny and illustration of the target structures from a desired angle. We stacked and edited the multi-plane microscope photos using the same software as for the macro photos (Figs 6, 8).

**Figure 4.**
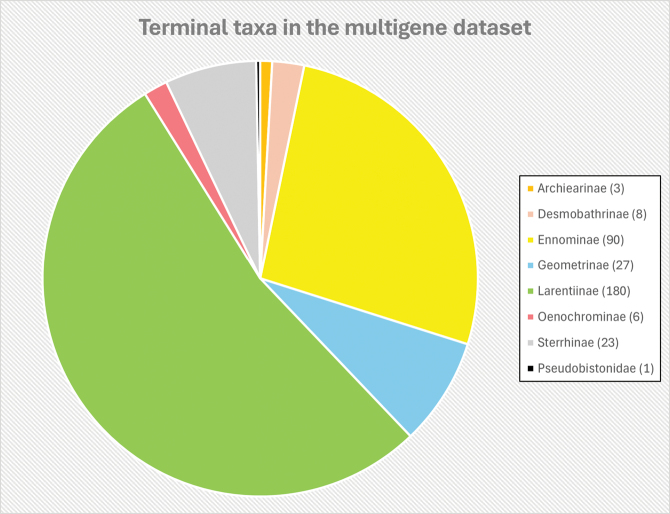
Breakdown of terminal taxa represented in the pruned and final phylogenetic tree, including the two new *Fynbosia* species.

Genomic DNA was extracted from leg tissues of the dry holotype specimens using the Qiagen DNeasy Blood and Tissue Kit following the manufacturer’s protocol at the Luomus DNA laboratory. DNA amplification and sequencing were carried out following the protocols we have described earlier (Englund et al. 2024). One mitochondrial gene (COI) and six protein-coding nuclear gene regions, Arginine Kinase (ArgK), sarco/endoplasmic reticulum calcium ATPase (Ca-ATPase), elongation factor 1 alpha (EF-1alpha), sorting nexin-9-like (Nex9), ribosomal protein (RpS5) and wingless (wgl) were successfully sequenced from the holotypes of *F. horingaria* sp. nov. (ME09) and *F. unicaria* sp. nov. (ME81). The sequences for each gene were aligned using the Muscle algorithm, and pairwise genetic distances were calculated using the Kimura 2-parameter model (Kimura 1980) for the COI gene (Suppl. material 3) in MEGA11 (Tamura et al. 2021). The aligned DNA sequences were then uploaded to VoSeq (Peña and Malm 2012). These sequences were combined with a multigene dataset published by Murillo-Ramos et al. (Murillo-Ramos et al. 2019) and pruned to a final dataset of 338 terminal taxa (Fig. 4), representing all subfamilies of Geometridae and an outgroup taxon (*Pseudobiston pinratanai* Inoue, 1994). The total length of the final dataset was 4980 bp, including gaps. GenBank accession numbers for the newly generated sequences are as follows: PV988405–PV988416.

The aligned gene sequences from VoSeq were downloaded and used for a maximum likelihood phylogenetic tree construction in IQ-TREE web server (v. 1.6.12) (Nguyen et al. 2015). We applied ModelFinder with ‘-m MFP + MERGE’ option for a best-fitting substitution model (Kalyaanamoorthy et al. 2017). The phylogenetic analyses were carried out using the edge-proportional ‘-spp’ option, which allowed each partition to have its own evolutionary rate. We applied ultrafast bootstrap (UFBoot2) approximations (Guindon et al. 2010; Hoang et al. 2018) using ‘-B 1000 -alrt 1000’ option to evaluate the node supports. To limit the risk of overestimating branch supports in ultrafast bootstrap approximation, we used the ‘-bnni’ option, which optimises each bootstrap tree using a hill-climbing nearest-neighbour-interchange search. The resulting trees we visualised and rooted in FigTree (v. 1.4.4) (Rambaut 2018) and modified the tree in CorelDraw (v. 25.1.0).

## Results

**Figure 5.**
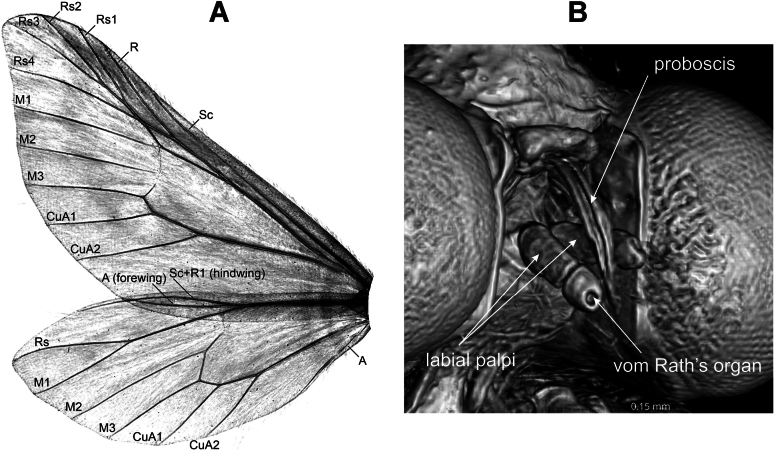
Micro-CT images of structural characters of *Fynbosia horingaria*. **A**. Wing venation; **B**. Head, ventral view.

The wing venation of *F. horingaria* sp. nov. (Fig. 5A) exhibits multiple features synapomorphic to the subfamily Larentiinae. Hindwing veins Sc + R1 and Rs are fused for a long-distance, forewing radial veins Rs1– Rs4 arise from the areole and are stalked, M1 proceeds in line with the anterior margin of the discal cell, and the sub-costal accessory cell between veins Sc and R is missing (Murillo-Ramos et al. 2021 and references therein).

The proboscis and the labial palpi, which comprise just two palpomeres (Fig. 5B), are vestigial, differing from those of most genera in Larentiinae. The cavity of vom Rath’s organ is present at the terminal tip of the labial palpus (Fig. 5B). A micro-CT 3D footage of the entire holotype of *F. horingaria* sp. nov. is shown in Suppl. material 1, and a close-up from the head exhibiting the proboscis and labial palpi in Suppl. material 2 from multiple view angles.

**Figure 6.**
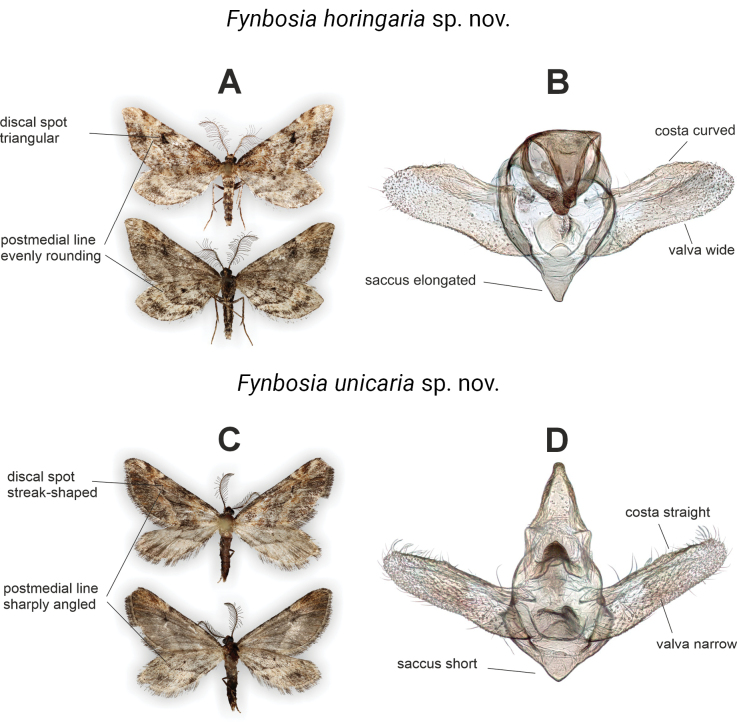
Key diagnostic characters of *Fynbosia* species. **A, C**. Wing ornamentation, dorsal view above, ventral view below; **B, D**. Male genitalia, caudal view.

The general appearance of the two *Fynbosia* species, wing shape, small size (Figs 3, 6) and resting position (Fig. 2D) would suggest an affiliation to the tribus Eupitheciini in Larentiinae (Viidalepp 2011), but the extremely long rami of the male antennae, the vestigial proboscis and labial palpi, and the male genitalia indicate otherwise. The male genitalia of *Fynbosia* are generally weakly sclerotised and contain few diagnostic features. The main diagnostic characters of the wing ornamentation and the male genitalia of *F. horingaria* sp. nov. and *F. unicaria* sp. nov. are highlighted in Fig. 6 and the keys below.

### Key 1 to species of *Fynbosia* gen. nov. based on male wing pattern

**Table:** 

1	Postmedial lines evenly rounded, best visible on the wing underside; forewing discal spot triangular (Fig. 6A)	***F. horingaria* sp. nov**.
–	Postmedial line sharply angled, forewing discal spot streak-shaped (Fig. 6C)	***F. unicaria* sp. nov**.

### Key 2 to species of *Fynbosia* gen. nov. based on male genitalia morphology

**Table:** 

1	Valva wide, length of valva less than three times the height of the valva, costa curved, saccus elongated (Fig. 6B)	***F. horingaria* sp. nov**.
–	Valva narrow, length of valva more than three times height of valva, costa straight, saccus short (Fig. 6D)	***F. unicaria* sp. nov**.

**Figure 7.**
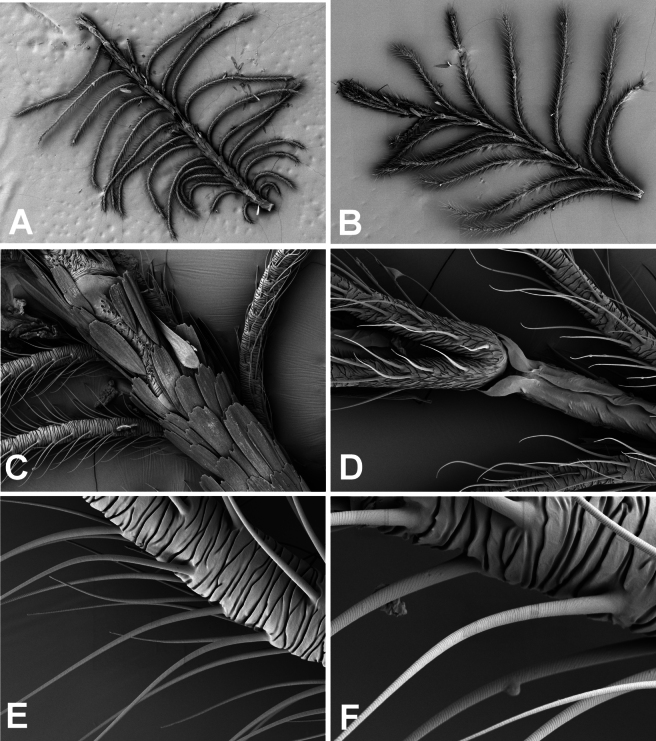
Scanning electron microscope (SEM) images of the antenna of *Fynbosia horingaria* sp. nov. **A**. Proximal half of the antenna, dorsal view; **B**. Distal half of the antenna, ventral view; **C**. Scale-covered flagella, dorsal view; **D**. Rami branching pairwise from the ventral side of flagellomere; **E**. Crumpled cutis of the ramus; **F**. Surface structure of the cilium. Magnification 250–3000×.

The male antennae of *Fynbosia* are disproportionately large and complex compared to those of most other genera in Larentiinae (Fig. 7). The extensive branching and cuticular folding (Fig. 7E, F) of the rami imply a highly sensitive male chemoreception system.

**Figure 8.**
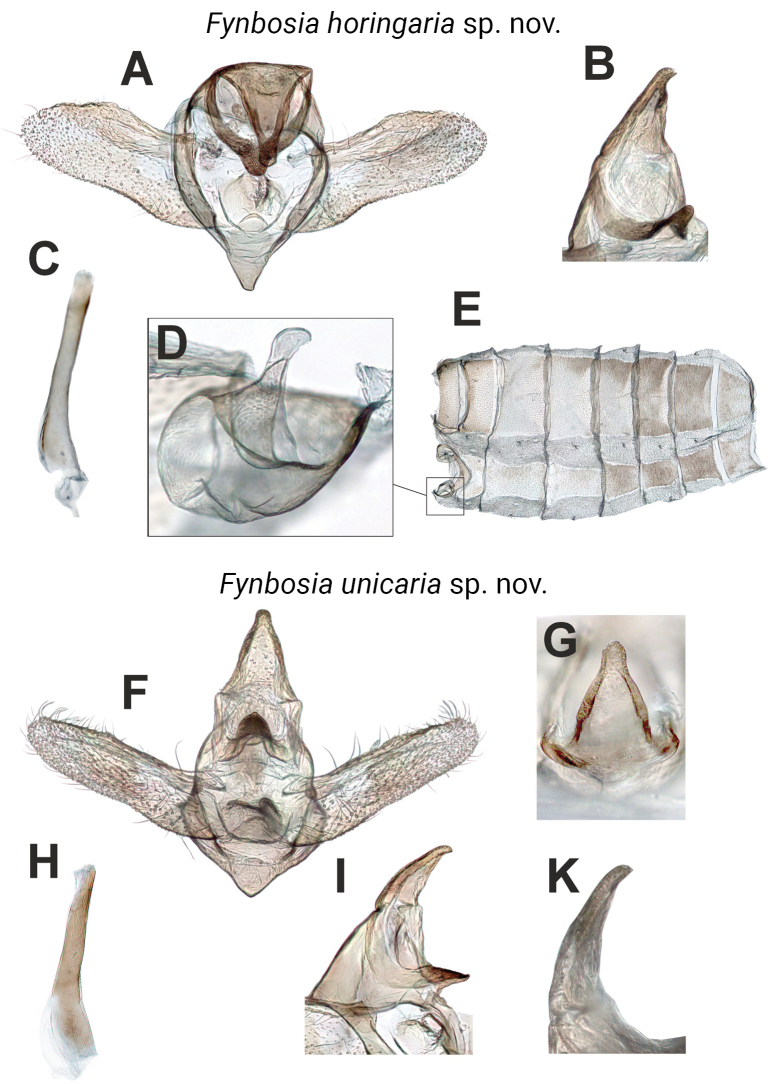
Dissected *Fynbosia* abdomens. **A**. *Fynbosia horingaria* sp. nov. (holotype) male genitalia, caudal view; **B**. Uncus, ventro-lateral view; **C**. Aedeagus; **D**. Tympanal organ; **E**. Abdominal skin; **F**. *Fynbosia unicaria* sp. nov. (holotype) male genitalia; **G**. Tip of uncus, dorsal view; **H**. Aedeagus; **I**. Uncus, ventro-lateral view; **K**. Tip of uncus, lateral view.

The male genitalia of *Fynbosia* are generally weakly sclerotised, exhibiting few diagnostic details (Fig. 8), yet the two known species can be distinguished by the shapes of the valva and saccus. We also attempted to render images of the male genitalia from the micro-CT scans, but in the absence of contrast-enhancing sample preparation methods applicable only to fresh specimens, we found the resulting images of insufficient quality to be presented here. The tympanal organs found on the ventral side of the abdominal segments 1+2 are rather small (Fig. 8E) with a slightly swollen tip of the ansa (Fig. 8D).

**Figure 9.**
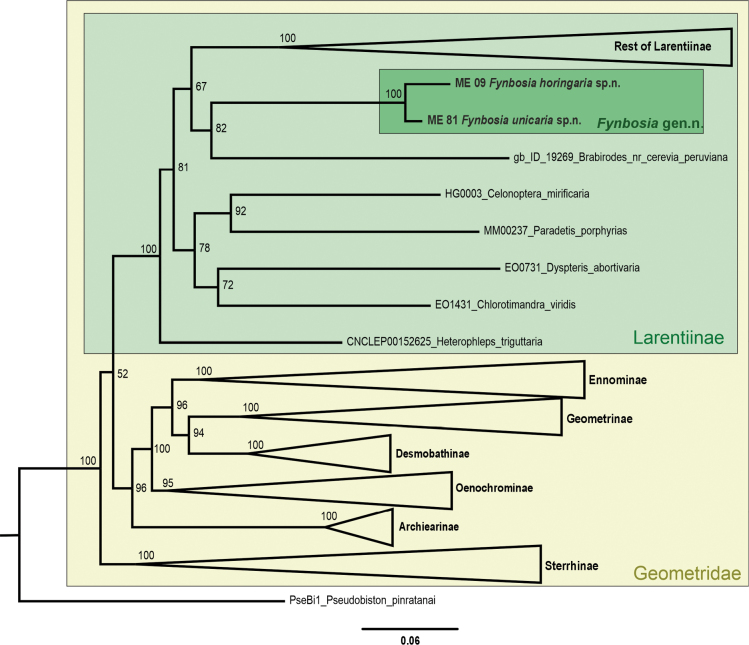
Multigene maximum likelihood phylogeny of the *Fynbosia* gen. nov., showing its placement within Geometridae.

The multigene molecular phylogeny (Fig. 9) supported our assessment based on morphological characters that *Fynbosia* moths in the family Geometridae and subfamily Larentiinae constitute an isolated lineage without close affiliation to any described tribus.

The COI genetic distance between the two *Fynbosia* species was 5%, and to their nearest neighbours (*Rheumaptera hastata* (Linnaeus, 1758) and *Xanthorhoe abrasaria* (Herrich-Schäffer, 1855)) in BOLD was more than 9%. The intraspecific genetic variance in *Fynbosia horingaria* sp. nov. (*N* = 3) is less than 1% (Suppl. material 3).

### Description of new taxa

#### Fynbosia

Taxon classificationAnimaliaLepidopteraGeometridae

Englund, Staude & Sihvonen gen. nov.

133DC6CD-3E41-5CE9-B9BB-56A878C73619

https://zoobank.org/4D24E751-A39F-4DA7-9116-B86EB49C52BC

##### Type species.

*Fynbosia horingaria* Englund, Staude & Sihvonen, sp. nov.

##### Diagnosis.

Southern African genus of subfamily Larentiinae (Lepidoptera: Geometridae) present at Cape Floristic Region not attributable or closely related to any described tribus of Larentiinae. Nocturnal small-sized moths with conspicuous male antenna with long ciliated rami, female antenna filiform. Vestigial, defunct proboscis and minuscule two-segmented labial palpi. Adult resting position, small size and elongated fore wing shape resembling those present in genera of Eupitheciini, but the structures of male antenna, proboscis, labial palpi, genitalia, and molecular phylogeny not compatible to any genera of that tribus.

Barcode (COI) 658 bp sequence minimum K2P distance to closest species (*Rheumaptera hastata* and *Xanthorhoe abrasaria*) > 9%. Closest sister lineage *Brabirodes* (Brabirodini) based on multigene 4980 bp maximum likelihood phylogenetic estimation.

##### Etymology.

Fynbos (> Afrikaans = fine bush) refers to the biome of Cape Floristic Region, where the type taxa reside.

#### Fynbosia horingaria

Taxon classificationAnimaliaLepidopteraGeometridae

Englund, Staude & Sihvonen sp. nov.

8C6C69E9-E369-591B-BEE0-C3BC82D0A2F1

https://zoobank.org/03D251B8-9E65-4F92-B168-71F803D317B5

##### Type material.

**Holotype** • ♂ (Figs 3A, 3C, 5 A, 6 A), South Africa: Calitzdorp, Swartberg, farm Zandrivier, 33.373°S, 21.330°E, 1200 m, 28 Apr. 2022, Mikael Englund leg., DNA sample ME09, gen. prep. PS 2872, micro-CT scan MS 2, coll. Ditsong Museum of Natural History, Pretoria, South Africa. **Paratypes** (5♂♂) • ♂ (Fig. 3E), South Africa: Western Cape, 40 km SE of Calitzdorp, farm Zandrivier, dry renosterveld – nama karoo 33.351°S, 21.317°E, 1200 m, 28–29 Apr. 2022, Mikael Englund, Pasi Sihvonen, Hermann Staude leg., DNA sample PS 1641, photo PMS_31002, coll. Englund, Finland; • ♂ (Fig. 3F), South Africa: Western Cape, 40 km SE of Calitzdorp, farm Zandrivier, dry renosterveld – nama karoo 33.351°S, 21.317°E, 1200 m, 28–29 Apr. 2022, Mikael Englund, Pasi Sihvonen, Hermann Staude leg., DNA sample PS 1659, photo PMS_31003, micro-CT scan MS 184, coll. Sihvonen, Finland; • ♂ South Africa: Western Cape, Calitzdorp, Seweweekspoort, Aristata, riverine fynbos, 33.4057°S, 21.4017°E, 12 Apr. 2023, A. Hofmann & J. Meineke, coll. Staude, South Africa; • ♂ South Africa: Western Cape, Calitzdorp, Seweweekspoort, Aristata, riverine fynbos, 33.4057°S, 21.4017°E, 12 Apr. 2023, A. Hofmann & J. Meineke, coll. State Museum of Natural History Stuttgart, Germany.

##### Diagnosis.

Adult male *F. horingaria* sp. nov. distinguishable from *F. unicaria* sp. nov. based on the wing patterns and genitalia. Both fore- and hindwing medial lines evenly rounded, not sharply angled near costa as in *F. unicaria* sp. nov., best visible on the wing undersides. Discal spots prominent, blackish, triangular in forewings (Figs 3, 6; Key 1). Male genitalia weakly sclerotized, but distinguishable from *F. unicaria* sp. nov. by the broader valva and elongated saccus (Figs 6, 8; Key 2). Average mtCOI genetic distance to *F. unicaria* sp. nov. is 5%.

##### Description.

**Adults**.

***Head***: Frons and vertex covered with light brown scales, mixed with some scattered dark brown scales, a string of hair-like scales protruding at the posterior margin of head, compound eye naked. Labial palpi short, with just two segments (Fig. 5B), length less than 1/5 of eye diameter, oriented ventrally, covered with yellowish ochreous scales; vom Rath’s organ cavity on the tip of the terminal palpomere. Proboscis vestigial, hardly visible between labial palpi, apparently defunct. Male antenna (Figs 3, 6, 7) bipectinate, flagella dorsally covered with ochraceous cells, flagellomeres light brown, except for the blackish swollen end of each flagellomere, ramus black, conspicuously long, length up to twice the diameter of compound eye, abundant long cilia covering the ramus. Female antenna filiform.

***Thorax***: Covered with mixed light to dark brown scales. Spur pattern on legs 0–0–2.

***Abdomen***: Segments covered with brown scales of varying tone, mottled with blackish scales. Some long hair-like scales extending distally from the proximal segments of the male abdomen. Bowl-shaped paired tympanal organs laterally in the segments 1+2, tip of ansa slightly swollen.

***Wings*** (Figs 3A, 3C, 5A, 6A): Forewing triangular, elongated, termen slightly convex from apex until midwing (M3), tornus round, without distinct transition from termen to dorsum. Wingspan 18–21 mm. Forewing ground colour ranging from light to medium brown with several dark wavy transverse lines most pronounced at costa. Proximal parts of medial and subcostal areas lighter brown than rest of forewing. Basal area with hazel brown tone. Discal spot distinct, triangular, blackish. Three dark brown blotches in terminal area, largest situated medially, smaller ones towards apex and tornus. Ante- and postmedial lines dark, evenly rounding inwards near costa in both fore- and hindwings. Hind wing apex rather sharply angled, discal spot dark. Terminal area lighter with darker suffuse plots forming discontinuous wavy line. Markings on hindwings more distinct on underside.

***Genitalia***: Male (Figs 6B, 8A–E): Genitalia generally weakly sclerotised with few diagnostic ornaments, except for uncus and gnathos. Valva wide, costa evenly rounded. Uncus rather well sclerotised, prominent with evenly downward hooking tip, juxta sclerotised with saddle-form upward hooking tip. Saccus elongated, aedeagus lacking cornuti.

Female genitalia unknown.

Immature stages unknown.

##### Genetic data.

GenBank accession numbers PV988405–PV988410.

##### Etymology.

Horing (> Afrikaans = horn); pertaining to the large and conspicuous, plume-like antennae of the male.

#### Fynbosia unicaria

Taxon classificationAnimaliaLepidopteraGeometridae

Englund, Staude & Sihvonen sp. nov.

D3AD5F36-BA32-5667-AFD9-145402272C63

https://zoobank.org/C8FD0470-84AE-4161-B1CC-E46208F9D241

##### Type material.

***Holotype*** • ♂ (Figs 3B, 3D, 6C), South Africa: Northern Cape, Calvinia, Hantamsberg, Van Rhynshoek, 33.377°S, 19.785°E, 1577 m, Renosterveld gonnabos, 31 Mar. 2022, leg. H.S. Staude, DNA sample ME81, gen. prep. PS 2919, micro-CT scan MS 125, coll. Ditsong Museum of Natural History, Pretoria, South Africa.

##### Diagnosis.

Adult male *F. unicaria* sp. nov. distinguishable from *F. horingaria* sp. nov. based on the wing patterns and genitalia. Both fore- and hindwing medial lines sharply angled near costa, not evenly rounded as in *F. horingaria* sp. nov., best visible on the wing undersides. Discal spots suffuse, dark, streak-like in forewings (Figs 3, 6; Key 1). Male genitalia weakly sclerotized, but distinguishable from *F. unicaria* sp. nov. by the narrower valva and short, blunt saccus (Figs 6, 8; Key 2). Average mtCOI genetic distance to *F. horingaria* sp. nov. is 5%.

##### Description.

**Adults** (male).

***Head***: Frons and vertex covered with light brown scales, string of hair-like scales protruding at posterior margin of head, compound eye naked. Labial palpi extremely short, length less than 1/5 of eye diameter, oriented ventrally, hardly visible between eyes, proboscis vestigial, defunct. Male antenna (Figs 3B, 3D, 6C) bipectinate, proximal third of flagella dorsally covered with dark brown scales, two distal thirds covered with ochraceous cells scattered with darker brown scales, ramus black, conspicuously long, length up to 1.5 times diameter of compound eye, abundant long cilia covering ramus.

***Thorax***: Covered with brown scales of varying tone. Spur pattern on legs 0–0–2.

***Abdomen***: Segments covered with brown scales of varying tone, mottled with blackish scales. Some long hair-like scales extending distally from proximal segments of male abdomen.

***Wings*** (Figs 3B, 3D, 6C): Forewing triangular, elongated, narrow, costa and termen almost straight, tornus round, without distinct transition from termen to dorsum, wingspan 20 mm. Forewing ground colour grey-brown, medial and terminal areas darker than rest of forewing, postmedial line almost straight, bordered distally with broad, suffuse hazel brown margin, turning inwards at sharp angle before touching costa. Discal spot stripe-like, black. Hindwings echoing forewing ornamentation pattern in less pronounced manner; postmedial line sharply angled, discal spot small, round, black. Dark, suffuse blotches in terminal area.

***Genitalia***: Male (Figs 6D, 8F–K): Genitalia generally weakly sclerotised and simple, except for the tip of gnathos. Few diagnostic features. Valva narrow, costa straight. Saccus short, borders almost straight, forming roughly right angle at tip. Aedeagus without well-sclerotised cornuti.

Female and immature stages unknown.

##### Genetic data.

GenBank accession numbers PV988411–PV988416.

##### Etymology.

*Unicus* (> Latin = sole, only); pertaining to the rarity and solitary known specimen of the species at the time of description.

## Discussion

There are close to 24000 described species of geometrid moths worldwide, and according to recent estimates, at least the same number of species remain undescribed (Rajaei et al. 2022). Within southern Africa, more than 2000 species of geometrid moths are present in extant collections, about one-third of which remain undescribed (Staude et al. 2025).

Against this background, the discovery of two new species from the poorly investigated fynbos is not particularly surprising. However, more surprising is that the new taxa do not fit well within the current classification system and apparently represent a phylogenetically isolated lineage. We consider our data adequate to support classifying *Fynbosia* in a monogenetic tribe within Larentiinae, but refrain from doing so because the taxon sampling underlying the current multigene molecular phylogenies is rather limited, particularly from the tropical areas. We expect the addition of new taxa will provide more robust support for the phylogenetic position of *Fynbosia* within the basal Larentiinae in the Geometridae tree of life.

We also examined the morphology of *Brabirodes peruviana*, the terminal taxon in our phylogeny (Fig. 9) as the closest relative to *Fynbosia* and found several morphological differences in *B. peruviana* compared to both *Fynbosia* species. For instance, the wing venation, male genitalia and tympanal organs in these species exhibit clearly distinct morphological features, as described in greater detail in a forthcoming publication (Sihvonen et al. 2025).

The stable geological and climatological conditions in the Cape region of South Africa have provided a unique setting for the floral and animal endemism in the Cape (Samways et al. 2024b). The elevated mountain slopes often support a flora distinct from the surrounding arid Karoo, rendering these biomes effectively isolated from one another. Especially phytophagous insect populations with likely limited dispersal, residing at higher elevations, such as *Fynbosia* moths, may be effectively allopatric.

## Conclusions

We experimented with the rendering of non-destructive diagnostic images from inner organs and structures from micro-CT scans (Englund et al. 2024) and found that we could produce adequate-quality images of wing venation, proboscis, and labial palpi, saving the rare specimens from further destruction. However, we assessed the micro-CT images of the tympanal organs and genitalia as insufficient for publication here due to weak natural sclerotization, and also to avoid destructive sample preparation methods.

While the life history, ecology and deeper phylogenetic relationships of *Fynbosia* remain largely unstudied at present, the new genus has some interesting traits, such as a vestigial proboscis, truncated labial palpi, and an apparently highly efficient male chemoreception apparatus, present in few other genera of the subfamily. The life history and tribal-level phylogeny for *Fynbosia*, as well as a few neighbouring genera, merit further study.

## Supplementary Material

XML Treatment for Fynbosia

XML Treatment for Fynbosia horingaria

XML Treatment for Fynbosia unicaria

## Additional information

### Use of AI

The initial draft of the abstract was generated based on the rest of the manuscript using the AI application Chat GPT (www.chatgpt.com).

### Author ORCIDs

Mikael Englund https://orcid.org/0009-0001-4778-9905

Elina Laiho https://orcid.org/0009-0004-6497-4178

Kyung Min Lee https://orcid.org/0000-0001-9055-1073

Hermann Staude https://orcid.org/0000-0002-2549-4372

Max Söderholm https://orcid.org/0009-0003-2877-1341

Pasi Sihvonen https://orcid.org/0000-0003-2237-9325

### Data availability

All of the data that support the findings of this study are available in the main text or Supplementary Information.

## Supplementary materials

Supplementary material 1 *Fynbosia horingaria* adult holotype 360° view Data type mp4 Explanation note Micro-CT video: *Fynbosia horingaria* adult holotype 360° view. https://binary.pensoft.net/file/1522627This dataset is made available under the Open Database License (http://opendatacommons.org/licenses/odbl/1.0/). The Open Database License (ODbL) is a license agreement intended to allow users to freely share, modify, and use this Dataset while maintaining this same freedom for others, provided that the original source and author(s) are credited. Mikael Englund, Elina Laiho, Kyung Min Lee, Hermann Staude, Max Söderholm, Pasi Sihvonen

Supplementary material 2 *Fynbosia horingaria* adult paratype Data type mp4 Explanation note Micro-CT video: *Fynbosia horingaria* adult paratype proboscis and labial palpi. https://binary.pensoft.net/file/1522628This dataset is made available under the Open Database License (http://opendatacommons.org/licenses/odbl/1.0/). The Open Database License (ODbL) is a license agreement intended to allow users to freely share, modify, and use this Dataset while maintaining this same freedom for others, provided that the original source and author(s) are credited. Mikael Englund, Elina Laiho, Kyung Min Lee, Hermann Staude, Max Söderholm, Pasi Sihvonen

Supplementary material 3 Genetic distances of *Fynbosia* and Larentiinae taxa Data type xlsx Explanation note Table: mtCOIa intra- and interspecific K2 genetic distances of *Fynbosia* and Larentiinae taxa: *Fynbosia* and 10 closest taxa (sheet 1, 14 taxa), Whole dataset (sheet 2, 332 taxa), and caption (sheet 3). https://binary.pensoft.net/file/1522629This dataset is made available under the Open Database License (http://opendatacommons.org/licenses/odbl/1.0/). The Open Database License (ODbL) is a license agreement intended to allow users to freely share, modify, and use this Dataset while maintaining this same freedom for others, provided that the original source and author(s) are credited. Mikael Englund, Elina Laiho, Kyung Min Lee, Hermann Staude, Max Söderholm, Pasi Sihvonen
